# Simulation of pandemics in real cities: enhanced and accurate digital laboratories

**DOI:** 10.1098/rspa.2020.0653

**Published:** 2021-01-27

**Authors:** A. Alexiadis, A. Albano, A. Rahmat, M. Yildiz, A. Kefal, M. Ozbulut, N. Bakirci, D. A. Garzón-Alvarado, C. A. Duque-Daza, J. H. Eslava-Schmalbach

**Affiliations:** 1 School of Chemical Engineering, University of Birmingham, Edgbaston, Birmingham B15 2TT, UK; 2 Faculty of Engineering and Natural Sciences, Sabanci University, 34956 Tuzla, Istanbul, Turkey; 3 Faculty of Engineering, Piri Reis University, 34940 Istanbul, Turkey; 4 School of Medicine, Acibadem University, lçerenköy, Kayışdağı 32, 34684 Istanbul, Turkey; 5 Department of Mechanical and Mechatronic Engineering, Universidad Nacional de Colombia, Cra 30 No 45-03. Bogotá, Colombia; 6 School of Medicine, Universidad Nacional de Colombia, Hospital Universitario Nacional de Colombia, Cra 30 No 45-03. Bogotá, Colombia

**Keywords:** epidemiology, COVID-19, numerical modelling, particle-based methods, discrete epidemiology

## Abstract

This study develops a modelling framework for simulating the spread of infectious diseases within real cities. Digital copies of Birmingham (UK) and Bogotá (Colombia) are generated, reproducing their urban environment, infrastructure and population. The digital inhabitants have the same statistical features of the real population. Their motion is a combination of predictable trips (commute to work, school, etc.) and random walks (shopping, leisure, etc.). Millions of individuals, their encounters and the spread of the disease are simulated by means of high-performance computing and massively parallel algorithms for several months and a time resolution of 1 minute. Simulations accurately reproduce the COVID-19 data for Birmingham and Bogotá both before and during the lockdown. The model has only one adjustable parameter calculable in the early stages of the pandemic. Policymakers can use our digital cities as virtual laboratories for testing, predicting and comparing the effects of policies aimed at containing epidemics.

## 1. Introduction

The epidemiological models used to predict the spread of infectious diseases are similar to the mathematical models used in chemistry. It is not a coincidence. They both describe the dynamics of populations evolving under the law of mass action (LMA). The underlying principle is that of ‘encounters between members’ producing an effect on the population. This effect can be a chemical reaction in a population of molecules or contagion in a population of humans. When population models are coupled with physical concepts like flux and conservation, they become the compartmental model. Classical epidemiological models are compartmental models with the flux being computed from the LMA.

The appeal of compartmental models lies in their simplicity. The complex dynamics of large numbers of molecules or individuals is condensed into a few ordinary differential equations. However, this simplicity comes at a cost. Standard epidemiological models do not account for space, behavioural aspects of the population or other external variables. These are introduced as case-specific adjustable coefficients that must be derived from data, making the model reliant on the timely availability of data. Moreover, every time the populations’ behaviour changes as a result of lockdown or other policies, these coefficients must be refitted to new data that will become available only after the policy is implemented.

To overcome the limitations of compartmental models, chemistry has moved beyond the LMA. Utilizing supercomputers, computational chemists today routinely simulate the trajectory and the collision of millions of atoms. This technique is called molecular dynamics (MD) and it is an irreplaceable tool in modern chemistry. Computational epidemiology, however, has not found its MD equivalent yet.

This study proposes a granular approach for modelling the spread of infectious diseases in real cities. Millions of individuals, their encounters and contagion are simulated at the individual level. We named this approach discrete epidemiology (DE) for its mathematical similarity to MD, discrete multiphysics [1] and, in general, particle methods.

Methodologically, DE results in a technique that combines, within an efficient computational framework, several approaches used in epidemiology. As compartmental models, DE divides the population into compartments, and, like stochastic models, it represents contagion as a Monte Carlo process. Similarly to census-calibrated models [2,3], it generates a digital population that, as in agent-based models (ABMs) [4,5], moves in a virtual city based on individual mobility plans (more details on the comparison between ABMs and DE is given in the next section). Also, as in human mobility models [6], it accounts for the unpredictability of human behaviour overlaying a time-dependent Wiener (or Lévy) walk on top of the mobility plan. DE is computationally very efficient because it takes advantage of algorithms, such as Verlet lists and massive parallelization, developed in over 70 years of MD. Today, MD can simulate billions [7] and even trillions [8] of atoms. This implies that, given the right amount of time and resources, DE can potentially simulate the mobility of the entire human population.

The paper is organized as follows: initially, we present several ‘toy models’. These introduce the DE theory step by step and, at the same time, validate the method against traditional epidemiological models. Each section introduces a new feature and is self-contained, meaning that it has its own independent ‘Methods’, ‘Results’ and ‘Discussion’. Finally, all toy models are combined to simulate real cities with millions of inhabitants. We recreate digital versions of Birmingham in the UK (1 million people) and Bogotá in Colombia (10 million people), replicating their geography and infrastructure (city limits, countryside, trains, bus lines, stations, etc.). Their virtual inhabitants have features coming from the statistics of the real population that are relevant to their mobility and susceptibility to the disease (age, residence, household size, employment, workplace, commuting, distance of contagion, infection probability, etc.). Once the digital city and its virtual population are generated, DE utilizes advanced hardware (high-performance and high-throughput computing) and software (massively parallel processing) to simulate the behaviour of all inhabitants in the city for several months. The position of every individual is updated every 60 s and when an infected individual encounters a susceptible one the probability of infection is handled stochastically. These computer-generated cities are then used as virtual laboratories to understand the unique features of the city, to determine the evolution of the disease within its boundaries and to test the effect of policies aimed at containing its spread.

### (a) Comparison between DE and ABMs

In its most general definition, agent-based modelling refers to the dynamics of a collection of agents following a set of predefined rules. If we consider the concept of ‘agent’ and ‘rule’ in their broadest sense, any discrete system, from MD to cellular automata and artificial neural networks, can be considered—somehow—an instance of an ABM. From this point of view, DE is no exception: it will always be possible to formulate a set of rules casting DE into an ABM.

Therefore, to frame the comparison between DE and ABMs in a meaningful way, we focus on how ABMs are commonly implemented in epidemiology. In particular, we frame the comparison in the context of the work carried out by Ajelli *et al.* [9], which, in turn, also compares different techniques.

Let us consider two extreme scenarios. In the first case, individuals move with random motion. The dynamics is unpredictable and determined, at each time step, by the generation of random numbers. An example is Brownian motion, which has been used to describe outbreaks in animal populations [10] and, in its simplest form, refers to our model in §2a. In the second case, we consider an ABM where individuals move according to mobility plans that are updated stochastically. After a link between two locations is established, the individual ‘jumps’ between these two locations.

In the first case, we describe the motion as a (random) trajectory since contagion can occur at each step of the individual trajectory. In the second, we describe the motion as ‘jumps’ since contagion can occur at the locations where individuals jump. DE combines both motions in different ways.

(i) Individuals follow mobility plans as in the ABM, but they do not jump from location A to location B. They follow a given path, which is also part of their mobility plan. However, the path is not the trajectory. In DE, individuals are pushed towards their path by the action of deterministic forces (see §2h), but the real trajectory is the result of these forces plus Brownian noise. Contagion can occur at any step of the trajectory. Two individuals can infect each other not only at home or at the workplace, but also if their trajectories coincidentally cross (i.e. the individuals are within the same contagion radius). Therefore, in DE, random contacts in the general population are calculated by ‘first principles’ (e.g. crossing trajectories). In ABMs, this is often regulated by probabilistic functions (e.g. eqn (3) in Ajelli *et al.* [9]), which introduce several additional adjustable parameters.
(ii) There are some hidden compartments in ABMs. Let us consider a trip from building A to building B. In ABMs, once individuals are inside the building, contagion is calculated on the basis of a ‘mixing rule’. In Ajelli *et al.* [9], for instance, contact with infectious members of the same household or workplace is based on homogeneous mixing. As a matter of fact, this makes every household and workplace in the model a compartment. In DE, individuals inside a building conserve their Brownian motion. This can be seen in §2d, where a fraction of the population moves into a gathering spot for several hours every day. Inside the gathering spot, we do not assume a homogeneous (or any other) social mixing rule and the probability of contagion is calculated by ‘first principles’ (i.e. presence in the same contagion radius).
(iii) Mobility plans work well for the people in employment or full-time study. Outside this group, motion is less predictable. In DE, the mobility of this part of the population is modelled as Brownian motion, as discussed in §2i.

From this point of view, DE inherits many concepts of ABMs but substitutes the concept of ‘jump’ with the concept of ‘path’ or ‘trajectory’. Consequently, DE can reuse many ideas from ABMs. For instance, in [11], contact and transmission rates were set to differ across distinct social contexts. The same concept can be implemented in DE. However, in DE, contact is based on proximity, which is resolved at the trajectory level. Therefore, the model must be parametrized only for transmission. This results in fewer adjustable parameters but comes at a price: Δ*t* of the simulation must be small enough to approximate the trajectory (here we use Δ*t* = 1 min), which makes the simulation computationally very intensive.

### (b) Nomenclature

Symbols used in this study are listed in table 1.

**Table 1.** Nomenclature.

*β* (s^−1^)	contact frequency
*γ* (s^−1^)	recovery rate
*γ*_*f*_ (s^−1^)	friction coefficient in equation (2.1)
*ξ* (kg m s^−2^)	random force in equation (2.1)
*A* (kg m s^−2^)	strength of the soft repulsive potential in equation (2.6)
*Dβ* (m^2^ m^−1^)	parameter in equation (2.2)
*f* ( − )	fraction of individuals with high mobility
*F*_REP_ (kg m s^−2^)	repulsion force in equation (2.1) and equation (2.7)
*F*_DRIFT_ (kg m s^−2^)	drift force in equation (2.1)
*F*_EXT_ (kg m s^−2^)	external force in equation (2.1)
*g* ( − )	radial distribution function
*L* (*m*)	reference length corresponding to 1 km in our simulation
*M* ( − )	instantaneous mobility defined in equation (2.5)
〈*M*〉 ( − )	time-averaged mobility
*N*_*S*_ ( − )	initial susceptible population
*N*_*I*_ ( − )	initial infected population
*N*_*R*_ ( − )	initial removed population
*p* (s^−1^)	probability of infection of an individual within *r* for the duration of Δ*t*
*r* (m)	radius of infection
*r*_*p*_ (m)	distance between particles in equation (2.6)
*r*_*c*_ (m)	cut-off distance in equation (2.6)
*s* ( − )	fraction of individuals with high mobility wearing a mask
*t* (s)	time
*T* (s)	reference time corresponding to 1 day in our simulation
*ν* (m s^−1^)	instantaneous particle velocity
〈*ν*〉 (m s^−1^)	time-averaged particle velocity

## 2. Methodology

### (a) A first, simple, discrete model

Individuals are represented by particles divided into susceptible (S), infected (I) and recovered (R). We use three groups, but the model can account for other categories commonly used in traditional epidemiological models (e.g. exposed or immune). Particles move in a two-dimensional domain D that represents the environment where the individuals live. In this first example, boundaries are periodic, i.e. particles that exit the domain on one side re-enter it from the opposite side. Random, human motion can be calculated with the Langevin equation [12]

2.1 $$m\frac{\text{d}\text{v}}{\text{d}t}=-m\gamma_{f}\text{v}+F_{\text{REP}}+F_{\text{DRIFT}}+F_{\text{EXT}}+\xi (t),$$

where *m* is the mass of the particle, **v** is the velocity and *γ*_*f*_ is a friction coefficient. *F*_REP_ is an interparticle repulsive force that prevents particles from overlapping. *F*_DRIFT_ keeps particles together when moving in a crowd. *F*_EXT_ refers to external forces preventing individuals from colliding with obstacles. *ξ*(*t*) is a fluctuating force accounting for the stochastic nature of human motion and is calculated at each time step by a random number generator with properties

2.2 $$\langle \xi (t)\rangle =0,\langle \xi (t)\xi (t+\delta t)\rangle =\frac{m^{2}D\gamma_{f}}{\Delta t^{2}}.$$

The parameter *D* is a measure of the fluctuation of the trajectory and is estimated for different human activities (e.g. indoors motion, outdoor activities, mass gatherings) from high-resolution GPS trajectories [13] or video recordings [14,15]. Equation (2.2) produces a Wiener process, i.e. a random walk where the mean squared displacement (MSD) is proportional to *t*. Lévy processes, where MSD is proportional to Δ*t*^*n*^ with *n* > 1, have also been suggested for human mobility [6]. For simplicity, we initially consider a Wiener process with *γ*_*f*_ → ∞. In this case, equation (2.1) reduces to a Brownian walk with *F*_REP_ → 0, *F*_DRIFT_ → 0 and *F*_EXT_ → 0. At each time step, the velocity of every individual is drawn from a normal distribution with mean *μ* = 0 and variance *σ*^2^ = 2*D*/Δ*t*, and its position updated accordingly.

**Figure 1.**
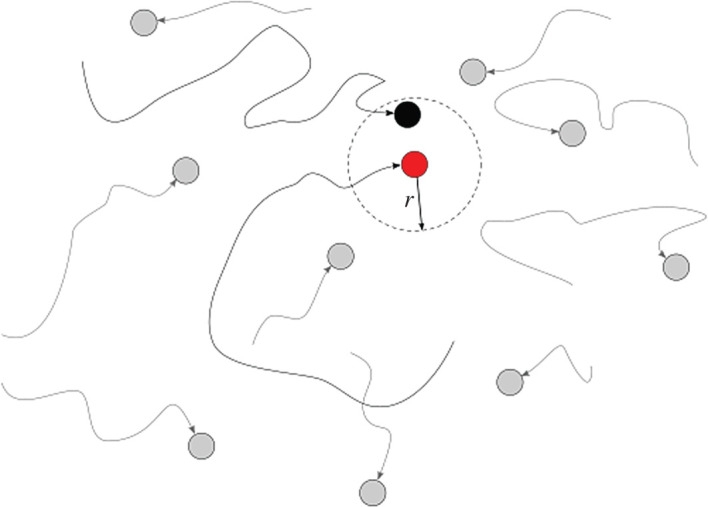
Motion and contagion of individual particles. (Online version in colour.)

Infected particles have a radius of influence *r*. Every time susceptible individuals move within the radius of influence of an infected one, there is an ‘encounter’ and the susceptible individual has a certain probability *p* of becoming infected (figure 1).

This probability is handled in a Monte Carlo fashion: if *p* is the probability that a contact lasting Δ*t* produces an infection, infection occurs if

2.3 $$R_{1}=p\Delta t,$$

where *R*_1_ ∈ [0, 1] is a random number with uniform distribution. The rate of recovery of infected particles is handled in a similar way. If *γ* is the recovery rate, an infected individual recovers if

2.4 $$R_{2}<\frac{\Delta t}{\gamma },$$

where *R*_2_ ∈ [0, 1] is another random number with uniform distribution. For details on Monte Carlo methods and the theoretical justification of equation (2.3) and equation (2.4), the reader can refer to [16].

**Figure 2.**
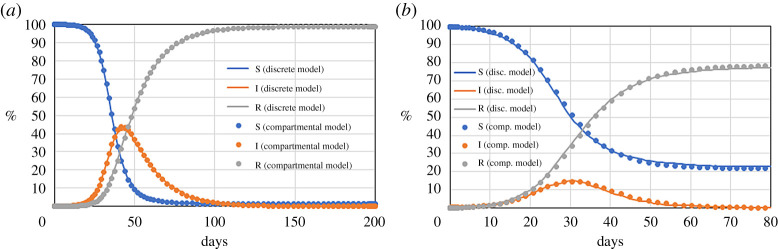
Comparison between the discrete model and the classical SIR model for two different cases: (*a*) *M* = 1.3, *r* = 4 m^−1^, *p* = 1/1000 min^−1^, which corresponds to *β* = 0.29 day^−1^ in the SIR model (*γ* = 1/15 day^−1^ for both models); (*b*) *M* = 5, *r* = 1 m^−1^, *p* = 1/30 min^−1^, which corresponds to *β* = 0.39 day^−1^ in the SIR model (*γ* = 1/5 day^−1^ for both models). (Online version in colour.)

Traditional compartmental models do not account for spatial inhomogeneities: every newly infected individual is automatically ‘spread’ over the entire domain. The DE model is not ‘perfectly mixed’ and individuals have positions that change over time. When their mobility is high with respect to the size of the domain, the model replicates traditional SIR models (figure 2). Mobility is defined by the dimensionless number velocity

2.5 $$M=\langle \nu \rangle \frac{T}{L},$$

where 〈*v*〉 is the average velocity of the individuals, *T* is a reference time and *L* is a reference length. Here, we use the size of the computational domain (1 km) for *L* and 1 day for *T*. Thus, *M* can be thought of as the average distance in kilometres travelled by an individual in a day. Both DE simulations in figure 2 are based on initial conditions *N*_*S*_ = 4069, *N*_*I*_ = 1 and *N*_*R*_ = 0. The time step used in the simulation is Δ*t* = 1 min.

**Figure 3.**
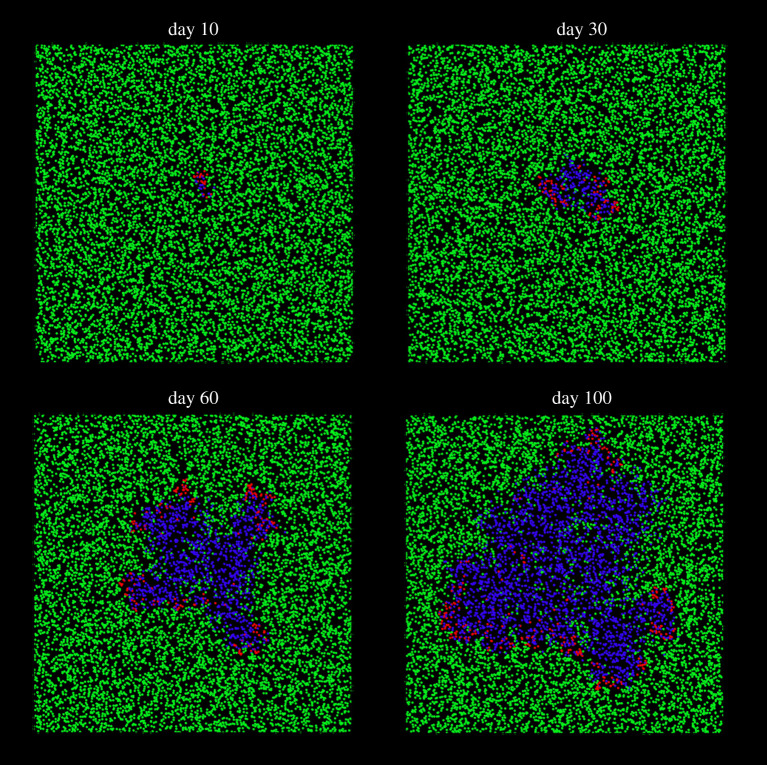
At low mobilities, the discrete model behaves like a lattice-SIR model: *M* = 0.05, *r* = 4 m^−1^, *p* = 1 min^−1^ and *γ* = 1/5 day^−1^; susceptible individuals are in green, infected individuals are in red, removed individuals are in blue.(Online version in colour.)

Figure 2 shows the temporal evolution of a population with high mobility; in this case, the DE model is equivalent to the ‘perfectly mixed’ SIR model. At low mobilities, individuals move slowly with respect to the size of the computational domain. In this case, the system is far from the perfect mixing assumption of the classical SIR model. The lattice-SIR model [17] accounts for this scenario: individuals do not move and are represented by nodes of a lattice that can infect their immediate neighbours. In DE, low values of *M* generate patterns typical of the lattice-SIR model (figure 3). The disease spreads along a front of infection, rather than being evenly distributed (electronic supplementary material, video 1).

Based on the particle mobility, this preliminary DE model replicates both the classic and the lattice-SIR model. We use this feature for modelling policies that limit a population’s mobility. Parameters like *r* (contagion distance), *p* (contagion probability of two individuals within r) and *γ* (recovery rate) are (at least in theory) intrinsic to the diseases and do not depend on the mobility of the population. Therefore, we consider scenarios where *r*, *p* and *γ* are constant and only *M* variable.

**Figure 4.**
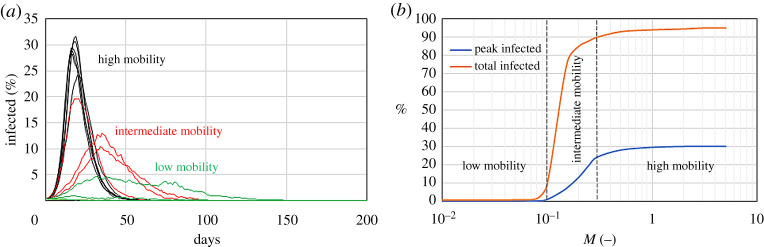
Effect of reducing the mobility of a system with *r* = 1 m^−1^, *p* = 1/30 min^−1^ and *γ* = 1/5 day^−1^ on the time series of the infected population (*a*) and the peak and total number of infected (*b*). Low-, intermediate, and high-mobility regimes correspond to *M* < 0.1, 0.1 < *M* < 0.3 and *M* > 0.3, respectively. (Online version in colour.)

In figure 4, it is possible to distinguish three different behaviours or regimes, according to the magnitude of *M*. If *M* > 0.3 (i.e. individuals travel an average distance from home of more than 3 km day^−1^), the system is perfectly mixed: the disease spreads at its full capacity and most of the population gets infected. If *M* < 0.1 (i.e. individuals travel an average distance from home of less than 1 km day^−1^), the system is segregated: pockets of diseases form, but, because of the low mobility, they do not easily spread to neighbour areas. Finally, if 0.1 < *M* < 0.3, an intermediate situation occurs. Figure 4 shows that there is a critical range that dramatically reduces the number of infections. As *M* goes from 0.3 to 0.1, the total percentage of the infected population decreases from 90% to 10%.

### (b) Second model: behavioural inhomogeneities in the population

In the previous model, the mobility reduction affects the whole population in the same way, which is not realistic. A fraction of the population must conserve higher mobility to ensure the functioning of society, and the entire population will not observe the quarantine with the same consistency. Standard epidemic models assume that all individuals behave in the same way, but DE models do not have this limitation. This second DE model divides the population into two groups: those who observe the lockdown and have low mobility, and those who do not observe the lockdown and maintain higher mobility. We assume that the population with high mobility has *M*_high_ = 0.3 and the population with low mobility *M*_low_ = 0.1. In the simulations, we vary *f*, the fraction of individuals with high mobility over the total population, and calculate the effect of *f* on the number of infected. The parameters that characterize the diseases are the same as the previous section (*r* = 1 m^−1^, *p* = 1/30 min^−1^ and *γ* = 1/5 day^−1^) with 20 initial infected individuals randomly distributed between the two populations.

**Figure 5.**
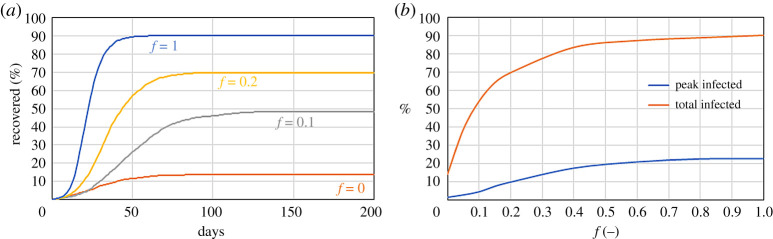
Effect of the fraction *f* of high-mobility individuals on a system with *r* = 1 m^−1^, *p* = 1/30 min^−1^, $M_{\text{\{low\}}}=0.1$, *M*_high_ = 0.3 and *γ* = 1/5 day^−1^ on the time series of the recovered population (*a*) and the peak and total number of infected (*b*). (Online version in colour.)

Figure 5 shows that the total number of infections strongly depends on *f*. It is enough that 10% of the population does not reduce its mobility to increase the number of total infected from 15% to 50%.

### (c) Third model: masks and social distancing

According to the literature, the efficiency of masks in reducing spreading is between 58% and 85% [18]. In the model, this is accounted for by a fraction of the populations with high mobility wearing the mask. We start with the example from §2b with *f* = 0.2 and consider that a fraction *s* of the *M*_high_ = 0.3 population wears a mask. Wearing a mask changes *p*. While in the previous example all individuals have the same *p*, in this case *p* is smaller for individuals wearing the mask. Assuming 70% mask efficiency: if *p* is the probability of infection without a mask, 0.3 *p* is the probability of infection when wearing the mask.

**Figure 6.**
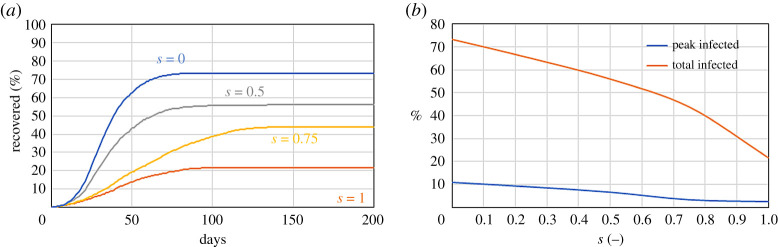
Effect of the fraction *s* of people with high mobility wearing a mask on a system with *r* = 1 m^−1^, *p* = 1/30 min^−1^, ${M}_{\textrm {low}} = 0.1\$, *M*_high_ = 0.3 and *γ* = 1/5 day^−1^ on the time series of the recovered population (*a*) and the peak and total number of infected (*b*). (Online version in colour.)

Figure 6 shows that masks can be effective, but the fraction of people with high mobility (i.e. interacting with other individuals on a regular basis) wearing them should be >50%. This is another ‘toy model’, but, despite its simplicity, it highlights some advantages of DE. The mask efficiency is retrieved from the literature and added to the model by ‘first principles’. We do not need to recalculate or reassess any parameter from new data as in traditional epidemiological models.

In the previous models, there is no limit on how close particles can approach each other. We can model social distancing with a repulsive force *F*_REP_ > 0 in equation (2.1). To achieve this goal, we introduce a potential known in MD as the ‘soft repulsive potential’

2.6 $$U=\left\{ \begin{array}{ll} A\left[1+\cos\left(\frac{\pi r_{p}}{r_{c}}\right)\right] & r_{p}<r_{c},\\ 0 & r_{p}\geq r_{c}, \end{array}\right.$$

where *r*_*p*_ is the distance between two particles, *r*_*c*_ is the cut-off distance and *A* is the rigidity of the potential. This potential produces a repulsive force

**Figure 7.**
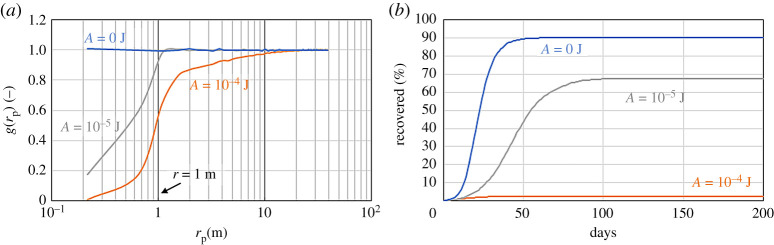
Effect of social distancing on a system with *r* = 1 m^−1^, *p* = 1/30 min^−1^, *M* = 0.3 and *γ* = 1/5 day^−1^: radial distribution function (*a*) and recovered population (*b*). (Online version in colour.)

2.7 $$F_{\text{REP}}=-\nabla U,$$

which tends to keep particles at a distance *r*_*p*_ > *r*_*c*_. We use *r*_*c*_ = *r*, which means that individuals try to keep a distance that is larger than the infection radius *r*. However, because the potential is soft, there are times when this does not occur. The lower the value of *A*, the softer the potential and the less likely it is for two particles to maintain the prescribed distance. In MD, atomic distances are shown by the radial distribution *g*(*r*_*p*_), i.e. the probability of finding a particle at a distance *r*_*p*_ from a given reference particle. Figure 7*a* shows *g*(*r*_*p*_) for different values of *A*. If *A* = 0, there is no repulsion, *F*_REP_ = 0 and *g*(*r*_*p*_) is flat, which means that all distances between individuals are equally probable. As *A* increases, it becomes less likely that two individuals will be found at a distance lower than *r*, which decreases the probability of contagion (figure 7*b*).

### (d) Fourth model: gathering spots (workplaces, schools, etc.)

In this section, we consider the presence of a gathering spot such as a workplace or a school in the domain.

**Figure 8.**
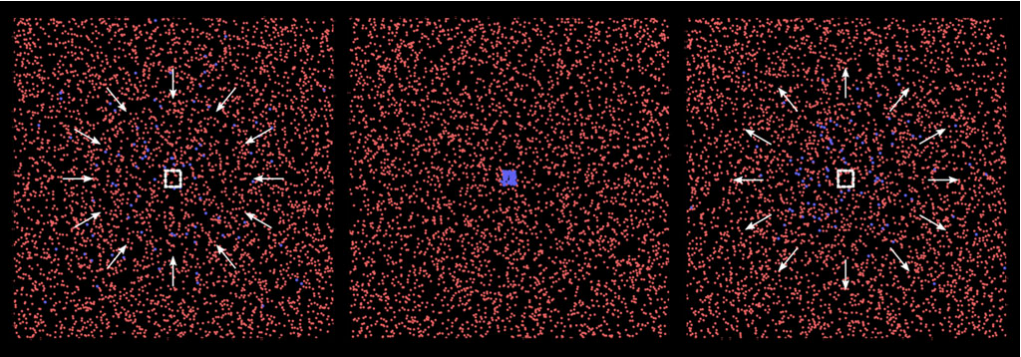
A fraction of particles is pushed towards a gathering spot in the centre of the domain for 8 h a day to model the presence of a school or a workplace. (Online version in colour.)

A periodic force *F*_EXT_ in equation (2.1) is used to push the particles inside a region into the centre of the domain (figure 8) and keep them there for 8 hours every day. After this time, their normal mobility (*M* = 0.1) is reintroduced. In this toy model, we have a single gathering spot, which is visited by 2% of the particles (blue particles in figure 8) randomly selected at the beginning of the simulation. The rest of the population has low mobility (*M* = 0.1) and does not visit the gathering spot unless they randomly move to the central area of the domain. All other simulation parameters are the same as in figure 4.

**Figure 9.**
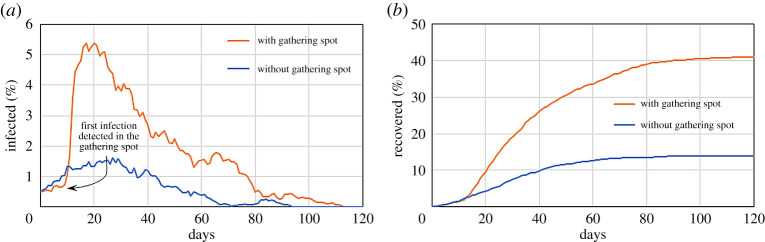
Effect of a gathering spot on a system with *r* = 1 m^−1^, *p* = 1/30 min^−1^, *M* = 0.3 and *γ* = 1/5 day^−1^ on the infected (*a*) and recovered population (*b*). (Online version in colour.)

Figure 9 shows the effect of the gathering spot on the infected population. During the first week, the effect looks beneficial. Part of the population gathers for 8 hours a day in the same spot. As long as no infection occurs here, the probability of contagion from outside is lower. However, after the first individual is infected, the rest of the population visiting the communal area follows suit. This produces a peak of infections that spreads to the entire population.

### (e) Fifth model: spatial inhomogeneities (city and countryside)

All models considered so far assume that the population density is the same in the whole domain. This is hardly the case as, for instance, cities have a higher population density than their countryside. The next toy model assumes that the population density in one region of the domain is higher than the rest.

**Figure 10.**
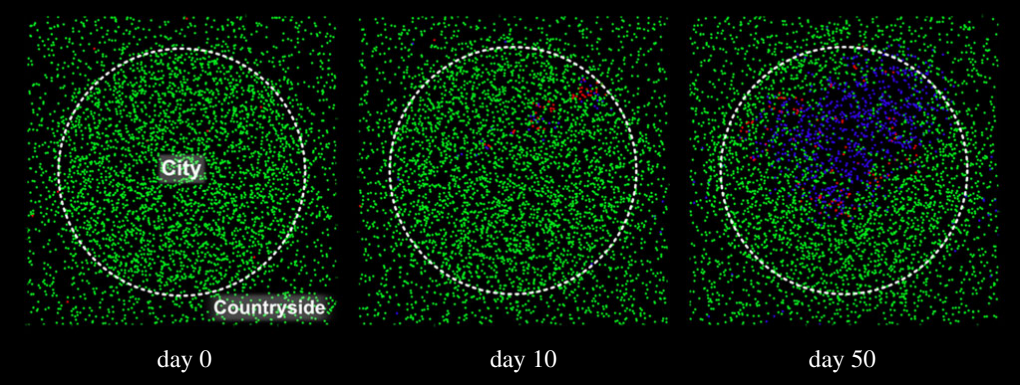
Evolution of a system with city/countryside density difference: *r* = 1 m^−1^, *p* = 1/30 min^−1^, *M* = 0.1 and *γ* = 1/5 day^−1^; susceptible individuals are in green, infected individuals are in red,removed individuals are in blue. (Online version in colour.)

In figure 10, the circle in the centre of the domain is the city; the rest is the countryside. The total number of particles in the domain is the same as in the previous examples, but they are distributed differently with the city having a population density double that of the countryside. Particles can cross the city boundaries, but an ‘invisible wall’ reflects some of them to maintain the population densities in the two regions at the prescribed values.

**Figure 11.**
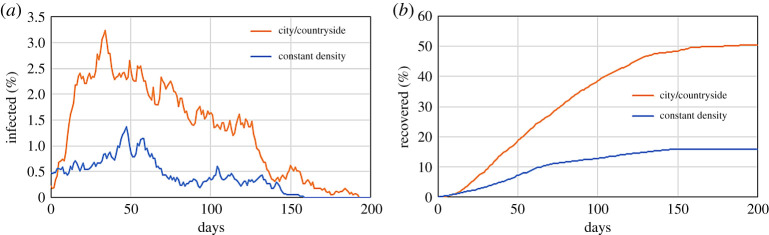
Effect of a population density difference between city and countryside on a system with *r* = 1 m^−1^, *p* = 1/30 min^−1^, *M* = 0.1 and *γ* = 1/5 day^−1^ on the infected (*a*) and recovered population (*b*). (Online version in colour.)

Figure 11 compares the result of this model with that of uniform density (the same as in figure 4). The presence of a region at higher density increases the total infections from 15% to 50%.

### (f) Sixth model: temporal inhomogeneities (day and night)

This model considers that individual mobility is lower at night. This is achieved by changing the mobility of the particles during the 24 hours. The velocity of the particles is always drawn from a Gaussian distribution with mean *μ* = 0 and standard deviation *σ* = *ν*. However, while in all previous models *ν* is constant, it now varies during the 24 hours according to

**Figure 12.**
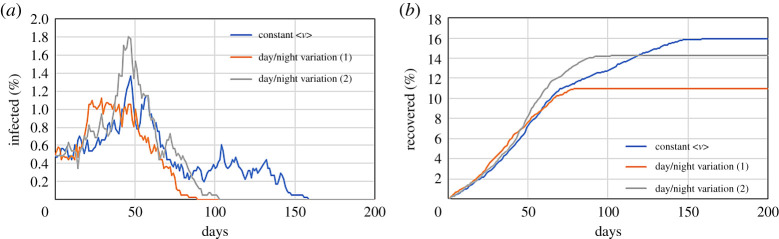
Effect of day/night mobility variations on a system with *r* = 1 m^−1^, *p* = 1/30 min^−1^, 〈*M*〉 = 0.1 and *γ* = 1/5 day^−1^ on the infected (*a*) and recovered population (*b*). (Online version in colour.)

2.8 $$\nu (t)=\frac{pi}{2}\langle \nu \rangle \left|\sin\left(\frac{\pi t}{T}\right)\right|,$$

where 〈*ν*〉 is the constant standard deviation used in all previous examples and the period *T* is equal to 1 day. Equation (2.8) guarantees that the average mobility during the 24 hours is the same as before. However, instead of being constant the whole time, it is maximal in the middle of the day and drops at night. Figure 12 compares time-dependent mobility (average 〈*M*〉 = 0.1) with the constant mobility (always *M* = 0.1).

Perhaps surprisingly, the simulation with day/night variation shows a lower infection. However, the stochastic nature of the model plays a role here. If the simulation is repeated with a different initial random distribution of infected particles, the results are somewhat different (grey line in figure 12). Technically speaking, all results (especially those at low mobilities) should be repeated several times with different random numbers to assess the probability of each outcome. Since the toy models are only meant to introduce the main features of DE, this is not carried out.

### (g) Seventh model: homes and families

**Figure 13.**
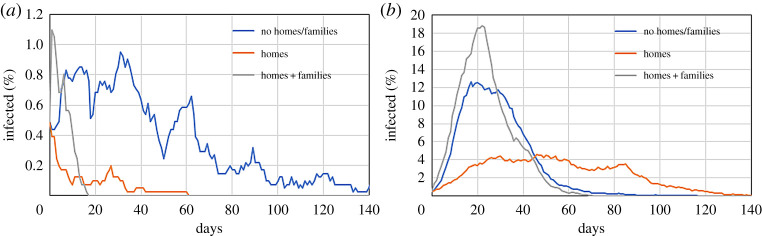
Effect on the infected population of the addition of homes and families in the model for a system with *r* = 1 m^−1^, *p* = 1/30 min^−1^ and *γ* = 1/5 day^−1^ in the cases of (*a*) 〈*M*〉 = 0.1 and (*b*) 〈*M*〉 = 0.3. (Online version in colour.)

So far, we have not considered people returning home at night. To account for this, we use an additional *F*_EXT_ in equation (2.1) that, between 20.00 and 04.00, keeps each particle at ‘home’ (the initial particle position). Conceptually, the approach is similar to that in §2b, but now *F*_EXT_ moves particles back to their individual homes rather than towards a gathering point. Ambiguities on the direction of ‘home’ may arise with periodic boundary conditions. Therefore, the box boundaries are switched to ‘reflective’. Particles moving outside the box by a certain distance are put back inside by the same distance with the sign of the corresponding velocity component flipped. Figure 13 shows that ‘homes’ with single occupants tend to suppress the infection. By returning to their initial position every night, particles visit a smaller portion of the domain. The model is extended to consider ’families’, i.e. multiple occupants sharing the same home. Several particles share the same initial position (just shifted a few metres to avoid overlapping) to reproduce a target average household size. Here, we assume a household size of 2.56 (Birmingham’s value). Families produce a different effect according to the mobility of the particles (figure 13). Low mobilities (〈*M*〉 = 0.1) result in an initial spike of contagions because people sharing the same house can easily infect each other. However, after that, the infection rate decreases sharply. In fact, by lumping particles together, the average distance between individuals belonging to different households increases, decreasing the probability of contact. However, high mobilities (〈*M*〉 = 0.3) compensate for the higher distance. In this case, the spike is not followed by a reduction in infections as for low mobilities.

### (h) Eighth model: public transport and streets

**Figure 14.**
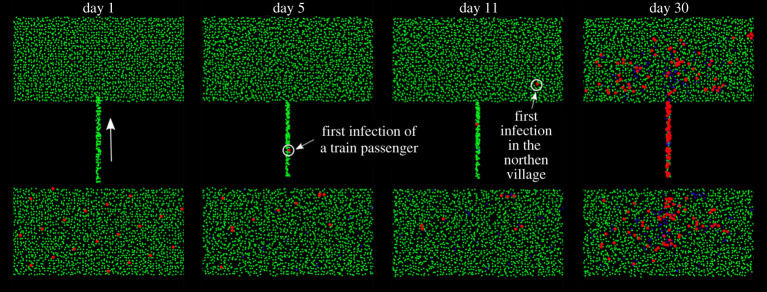
Evolution of two separated systems with 200 commuters moving between two regions: *r* = 1 m^−1^, *p* = 1/30 min^−1^, 〈*M*〉 = 0.1 and *γ* = 1/5 day^−1^; susceptible individuals are in green, infected individuals are in red, removed individuals are in blue. For graphical reasons, the size of the dots representing infected individuals is eight times larger than that of susceptible and recovered individuals. (Online version in colour.)

In this model, we consider two separate regions of 2000 particles separated by an empty zone. For reference, we call the upper region the ‘northern village’ and the lower region the ‘southern village’. Particles have variable day/night mobility as in §2f and return home at the end of the day as in §2g. All particles infected at *t* = 0 are in the southern village (figure 14). If there is no connection between the two regions, and since particles have low mobility (〈*M*〉 = 0.1), the epidemic lasts 20 days and only a small fraction of the population of the southern village is affected. Since the two villages are separated, the infection has no mean of spreading to the northern village.

The previous scenario is modified by assuming that the two villages are connected by rail. ‘Home’ and ‘workplace’ positions of each particle are selected randomly at the beginning of the simulation. Two hundred particles from the southern village are allocated to the group ‘commuters’. Their home is in the southern village and their workplace in the northern village. Every morning, commuters move to the northern village and go back home during the evening by train. Mathematically, the collective movement of individuals using the train is modelled as a force field (a space-dependent *F*_EXT_ in equation (2.1)) that directs a fraction of the southern population to the north in the morning and then back to the south in the evening. In the case under investigation, this force field is expressed as

2.9 $$F_{\text{EXT}}(x,y)=(-xF_{x},F_{y}),$$

where *f*_*x*_ and *f*_*y*_ are determined to set the travelling time to the wanted value (1 hour in this example). Equation (2.9) constrains particles to follow a given path (the train line), and it is only applied for the duration of the outward journey. After the particles arrive in the northern village, the force field is substituted by an *F*_EXT_ that moves the particles to their workplaces, as in §2d. Once particles reach their workplace, for the next 8 h, no additional force is applied, and they move based on their background mobility (〈*M*〉 = 0.1). At the end of the working day, equation (2.9) is applied in the opposite direction to simulate the return journey. Finally, an additional force drives the particles home for the night, as in §2d. Electronic supplementary material, video 2 shows the evolution of the infection in detail.

**Figure 15.**
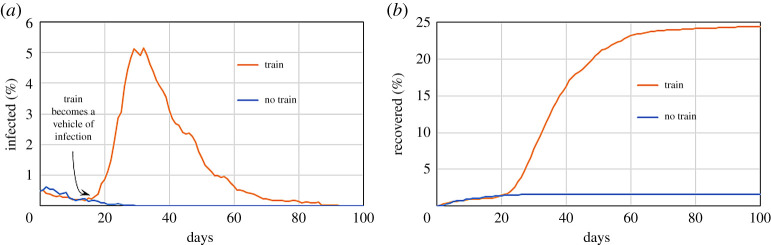
Effect of 200 commuters moving between two regions on a system with *r* = 1 m^−1^, *p* = 1/30 min^−1^, 〈*M*〉 = 0.1 and *γ* = 1/5 day^−1^ on the infected (*a*) and recovered population (*b*). (Online version in colour.)

The presence of the train increases dramatically the spread of the disease for two reasons. Firstly, passengers in the train are at close contact, increasing the chances of infection. Secondly, they bring the infection to the other village. Figure 15 compares the scenarios with and without train passengers.

**Figure 16.**
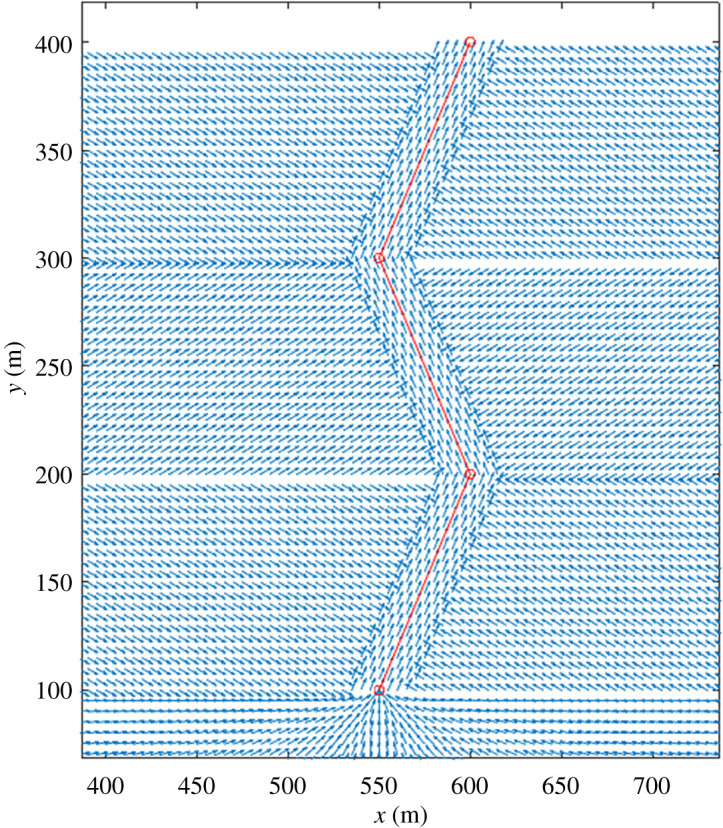
Example of a ‘force field’ used to constrain particles within a given path or street (red line).(Online version in colour.)

A similar idea can be used to model the presence of buses in the city. Commuters that use a specific bus line are subjected to time-dependent force fields (e.g. figure 16) that constrain them to follow a given path. The force field is only used for commuters that use public transportation and find themselves at close contact with other passengers. Buses are discussed in detail when modelling Bogotá.

### (i) Putting all together 1: simulating the city of Birmingham, UK

This section puts together all ideas developed so far to simulate Birmingham in the UK (∼1 million inhabitants). The first step is to digitally recreate the area of the city and its boundaries framed in a computational box of side 30 km. The area between the city and the box is the countryside. Boundary conditions at the box are ‘reflective’, as discussed in §2g. Particles are free to move from the city to the countryside and vice versa.

After the geometry is set, the next step is to generate its virtual population. The population density of the city is 3649 inhabitants per km^2^, while the surrounding countryside has 456 inhabitants per km^2^. For simplicity, density differences within the city are ignored. This generates 1 072 924 particles, each representing a digital inhabitant: 960 483 inside the city and 112 441 in the countryside. Particles are arranged on a randomly perturbed lattice. The initial location of each particle is labelled as ‘home’ and the average household size is 2.56 [19], as discussed in §2g. In theory, we could use the exact locations of all households in Birmingham, but this is left for future versions of the model.

The third step consists in generating the individual properties of each particle affecting either its mobility or susceptibility to the disease. An age is randomly attributed to every individual according to the statistical age distribution in the UK [20]. On average, an individual in the UK walks around 1 km day^−1^ [21]. This mobility is accounted for as a random walk, as in §2a. The population is further divided into employed, pupils and unemployed. Individuals are randomly allocated to these groups according to employment statistics based on age groups [22], as shown in table 2. Individuals between 6 and 16 years of age are considered to be pupils; individuals above 64 unemployed.

**Table 2.** Percentage of the employed population in the UK within age groups.

age group (years)	16–24	25–34	35–49	50–64
% of employed	74.5	84.3	85.4	72.7

**Figure 17.**
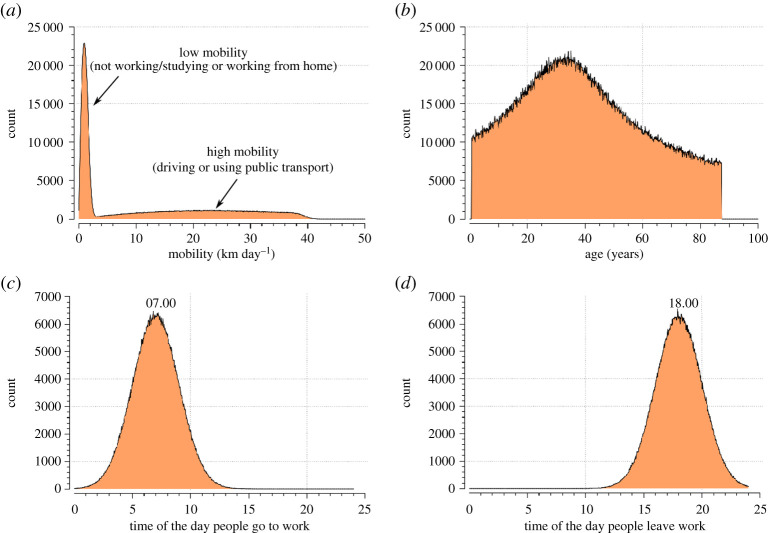
Some of the statistics of the virtual population used in the simulations: (*a*) mobility, (*b*) age, (*c*) time of the day when people go to work and (*d*) time of the day when people leave work. (Online version in colour.)

For each member of the employed and pupil group, a location on the map is assigned as, respectively, a workplace or school. At this stage, these locations are random: the painstaking task of specifying the exact locations of all workplaces and schools in Birmingham is left for future versions of the model. During the day, the particle goes to this location (as discussed in §2d) and returns home in the evening (as discussed in §2g). This additional mobility is applied only for the time required to reach its destination and is added on top of the 1 km day^−1^ mentioned before. Since 10% of the working population works from home [23], this mobility bonus is not given to 10% of the employed group selected randomly. According to statistics, the average distance of an individual from work is 10 km [24] and of a pupil from school is 3 km [25]. Distances between home and work and home and workplace are randomly assigned to each individual according to these statistics. The time of day when the commute from and to work occurs is different for each individual and allocated randomly based on the distributions shown in figure 17*c*,*d*. We could not find precise statistics for these times, which, therefore, are based on common sense.

The probability of interacting with other individuals is higher if the commute occurs by means of public transportation. Several ‘nodes’ (e.g. bus, train stations) where commuters congregate are created on the map. Two-thirds of the working population use these nodes [26]; the remaining one-third drive to work and, therefore, do not transit through these nodes. Future versions of the model could account for the exact places of all nodes (e.g. bus, train stations); in this study, for simplicity, 300 nodes are created at random locations.

Based on the description above, at any given time, an individual can have either low mobility, which represents an individual staying at home or walking near home (average distance 1 km day^−1^), or high mobility, which represents a particle commuting to work (average distance 10 km day^−1^) or school (average distance 3 km day^−1^). However, according to the statistics [27], during the day, there are 400 000 vehicles on Birmingham’s streets. While employed individuals are at work and pupils at school, the mobility of a fraction (chosen randomly) of the remaining population is increased to 10 km day^−1^ to simulate people driving for reasons other than commute (e.g. shopping, leisure). This additional mobility is added as a random walk (§2a) and considering day/night differences (§2f).

Besides the city and its population, we need to model the disease. Here, we use *r* = 2 m and *γ* = 1/7 day^−1^, which have been suggested for coronavirus (COVID-19) [28,29]. The parameter *p* is used to adjust the model’s response when no policy against the epidemics is implemented. It accounts for features that are not directly considered in the model and must be ‘captured’ from real data. This includes the probability that infected individuals are identified, and, therefore, it also depends on the quantity and quality of testing carried out in the city. We adjust the parameter *p* to fit the initial Birmingham data before the lockdown. The value *p* = 5 × 10^−3^ min^−1^ gives the best fitting, which corresponds to a reproduction number *R*_0_ = 2.4 as calculated for Birmingham during the initial phase of the infection [30]. Seasonal variation of the infection rate is not considered at this stage.

The final step consists in running the simulations that cover 200 days with a time resolution of 1 min. They are carried out with the software LAMMPS (Large-scale Atomic/Molecular Massively Parallel Simulator) [31], an MD program that can be repurposed for generic particle-based simulations. In terms of hardware, we use BlueBEAR, the University of Birmingham’s supercomputer for high-performance computing (HPC) and high-throughput computing. Each simulation uses 120 dual IBM POWER9 CPUs. For 1 million particles, the duration of each simulation is between 1 and 2 h.

**Figure 18.**
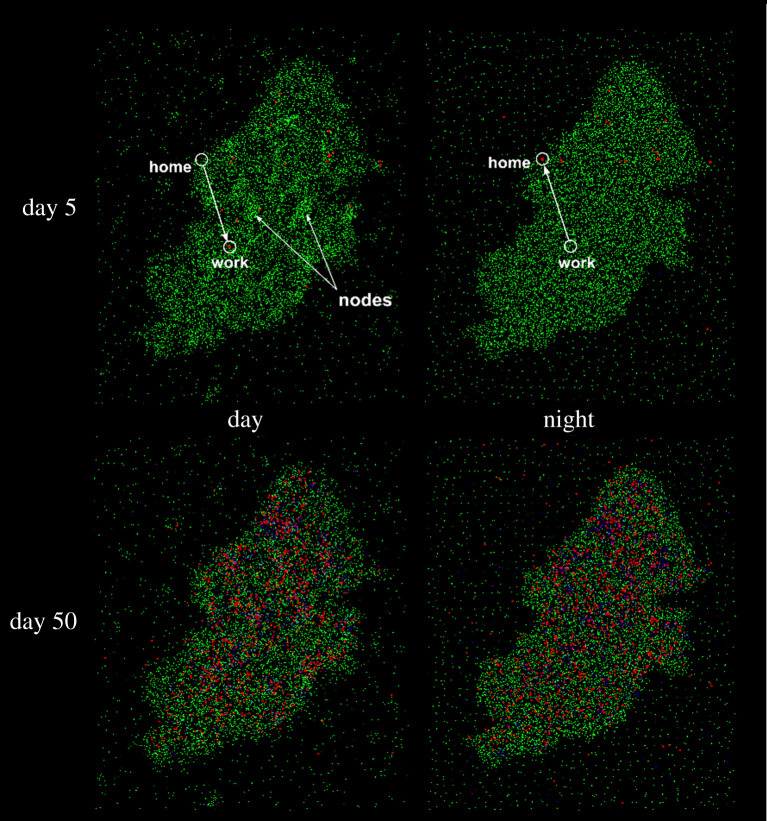
Infection dynamics in the virtual Birmingham and its surroundings. For graphical reasons, only 1% of the population is represented and the size of the dots representing infected individual is 10 times larger than that of susceptible and recovered individuals. An example of home–work commuting and a few ‘nodes’ are highlighted. (Online version in colour.)

**Figure 19.**
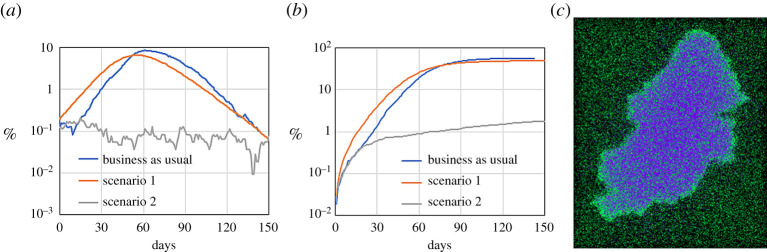
Time evolution of (*a*) infected and (*b*) recovered populations for three Birmingham scenarios; (*c*) spatial distribution of susceptible and recovered populations for the ‘business as usual scenario’ after 200 days (only 1% of the particles is shown). (Online version in colour.)

Figures 18 and 19 show the spatial and temporal evolution of the disease in the virtual Birmingham for the ‘business as usual scenario’. Electronic supplementary material, video 3 shows the particles’ mobility for 24 h: every second of the video represents 40 real minutes; particles with high mobility have a lighter colour. The video also shows the trip from home to work of a single particle in yellow. Electronic supplementary material, video 4 shows the evolution of the infection: every second of the video represents 2 days. In both videos, for graphical reasons, only 1% of the particles are shown. Figure 19*a*,*b* shows the overall evolution of the infected and recovered population. Figure 19*c* shows that, at the end of the epidemic, the city is hit harder than the countryside.

**Figure 20.**
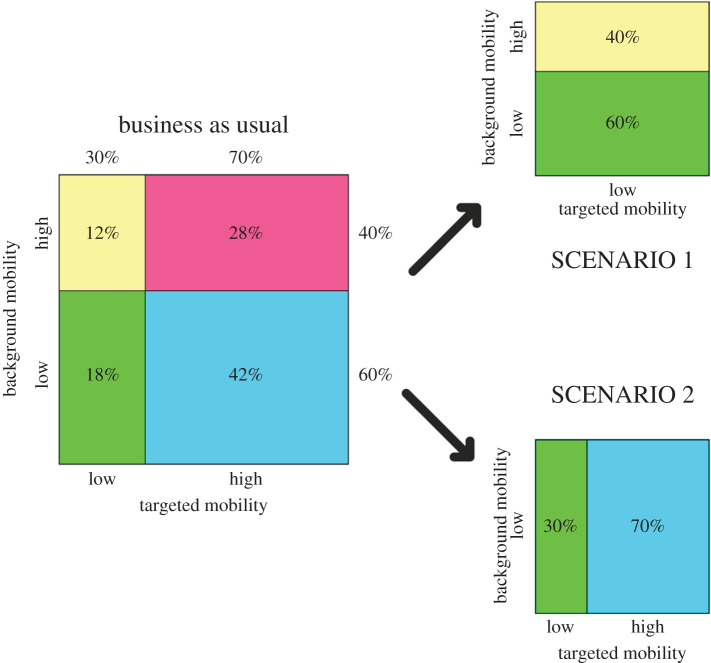
Three scenarios for the city of Birmingham. (Online version in colour.)

We can distinguish two factors that determine how the infection spreads within the city. The first is the ‘targeted mobility’ that accounts for time-recurring trips to specific locations (e.g. school, work, train stations), which, mathematically, is simulated with directional forces (§2d) or force fields (§2h). The second is the ‘background mobility’ that accounts for trips that do not occur every day and are not predictable *a priori* (e.g. shopping, leisure), which are simulated by random walks (§2a). In the ‘business as usual’ scenario, individuals can be roughly divided into four groups according to the level of their background and targeted mobilities (figure 20). The model can be used to evaluate the relative importance of the two mobilities on the spreading of the infection. This can be achieved by comparing the ‘business as usual’ scenario with two other ‘extreme’ scenarios (figure 20). In scenario 1, the individuals conserve their background mobility from the ‘business as usual’ scenario, but the targeted mobility is completely removed. In this scenario, there are no restrictions to the population movements, but all workplaces, schools and public places are closed. Scenario 2 is the opposite: the background mobility is reduced to a minimum (0.1 km day^−1^), but the targeted mobility is the same as the ‘business as usual’ scenario. In this case, workplaces and public places are regularly open, but, except for travelling to these places, the population’s mobility is severely restricted. These are extreme and somehow unrealistic scenarios but are useful for weighing the respective roles of the background and targeted mobilities. Figure 19 compares the infected and recovered populations of scenarios 1 and 2 with the ‘business as usual’ scenario. Both scenarios decrease the total number of infections but reducing the background mobility is more effective. This can be explained by the conceptual difference between targeted and background mobility. Targeted mobility is predictable: it accounts for individuals going always to the same places (e.g. home and workplace) and meeting always with the same people (e.g. family members and colleagues). Therefore, the pool of potential interactions is limited. In contrast, background mobility has a potentially unbounded stochastic component. In theory, a particle moving only with background motion can approach and infect any other particle in the computational domain and, therefore, its spreading potential is higher.

**Figure 21.**
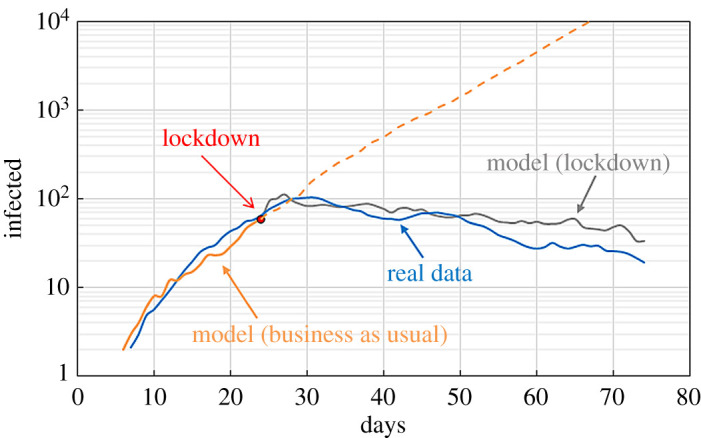
Comparison between real data (7 day moving average) and model output. (Online version in colour.)

Figure 21 shows how the model performs against real data of the COVID-19 infection in Birmingham by comparing the real data with the ‘business as usual’ scenario initialized with five initial infected individuals randomly distributed in the population. Real data are shown as a 7 day moving average.

The ‘business as usual’ model correctly simulates the data until the day of lockdown. This is not surprising because the parameter *p* was chosen to fit the real data. What makes the DE model superior to traditional epidemiological models is the handling of the lockdown. During the lockdown, both the targeted and the background mobility of the population are reduced. Google made available ‘mobility reports’ that show movement trends (based on requests for directions in Google Maps) over time by geography, across different categories of places such as retail and recreation, groceries and pharmacies, parks, transit stations, workplaces and residential [32]. We can use these data as an estimate of the reduction of mobility during the lockdown. The Mobility Report for the West Midlands (Birmingham’s metropolitan county) indicates that the drop in mobility during the lockdown for ‘retail and recreation’ and for ‘workplaces’ was, respectively, 79% and 58%. We feed these data into the model by reducing the background mobility and the targeted mobility by the same amount. This is achieved by reducing by 79% the number of individuals with high background mobility (10 km day^−1^) and by 58% the numbers of individuals going to work every day; additionally, schools are closed and all trips to and from schools are cancelled. These modifications are applied after day 23; so that the simulation follows the ‘business as usual’ model until the day of the lockdown and the ‘lockdown model’ after its implementation. Figure 21 shows that the simulation compares well with the real data. This agreement is achieved with only one adjustable parameter, *p*, which can be considered as a property of the city and estimated at the beginning of the infection. Since *p* is decoupled from the mobility, it does not change when the lockdown is implemented. Therefore, the model can predict the effect of the lockdown only based on ‘first principles’. That is, features that are measurable (e.g. with the Mobility Reports) and have a direct connection with the intended target of the lockdown policy (e.g. the population drop in mobility).

**Figure 22.**
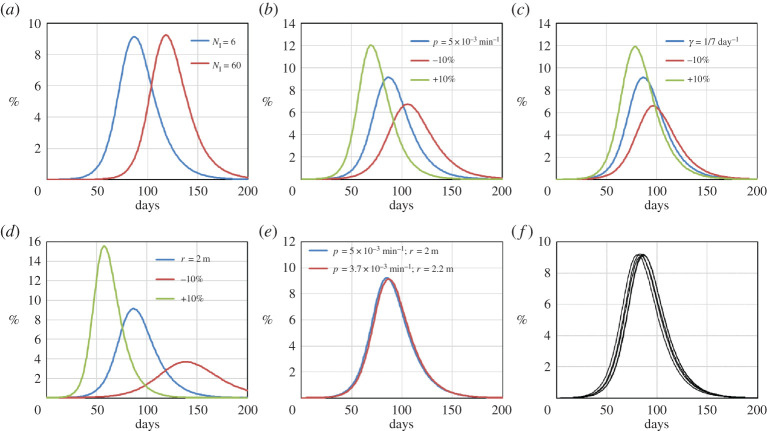
Sensitivity and statistical analysis of the Birmingham model of the percentage of infected individuals with time. Effect of the initial number of infected individuals *N*_I_ (*a*); effect of a ±10% change in *p* (*b*); effect of a ±10% change in *γ* (*c*); effect of a ±10% change in *r* (*d*). Change of the free parameter *p* to compensate for an error in *γ* (*e*). Seven simulations are carried out with different seeds of the random number generator (*f* ). (Online version in colour.)

To show how uncertainties in the input parameters affect the results, (i) we check the sensitivity of the results to the model’s parameters that model contagion (i.e. *N*_I_, *p*, *γ* and *r*) and (ii) we perform a statistical analysis of the model by running the same simulation with different seeds of the random number generator. In this way, we can evaluate several stochastic outcomes of the same simulation. Figure 22*a* shows the effect of the initial number of infected individuals on the simulation. The profile is similar, and the main difference consists in a temporal shift between the two profiles. We also evaluate the effect of a ±10% change in the infection probability *p* (figure 22*b*), the recovery rate *γ* (figure 22*c*) and the radius of infection *r* (figure 22*d*).

To quantify the sensitivity of the results to these parameters, we define the relative error *ϵ* as

2.10 $$\epsilon_{x}=\frac{\Delta I_{MAX}}{\Delta x},$$

where *x* indicates the parameter *p*, *γ* or *r*, Δ*x* is the percentage change of *x* and Δ*I*_*MAX*_ is the change in the percentage of infected individuals at the peak of infection resulting from Δ*x*. Table 3 shows two values of *ϵ*_*x*_: $\epsilon_{x}^{+}$ corresponding to Δ*x* = +10% and $\epsilon_{x}^{-}$ corresponding to Δ*x* = −10%. The third column shows 〈*ϵ*_*x*_〉, which is the average between $\epsilon_{x}^{+}$ and $\epsilon_{x}^{-}$.

**Table 3.** Values of $\epsilon_{x}^{+}$, $\epsilon_{x}^{-}$ and 〈*ϵ*_*x*_〉 for the parameters *p*, *γ* and *r* for the Birmingham model approximated to the second decimal place.

	$\epsilon_{x}^{+}$	$\epsilon_{x}^{-}$	〈*ϵ*_*x*_〉
*ϵ*_*p*_ (min)	0.24	0.29	0.27
$\epsilon_{\gamma }$ (day)	0.25	0.28	0.26
*ϵ*_*r*_ (m^−1^)	0.54	0.64	0.59

The error 〈*ϵ*_*x*_〉 tells us how much the percentage of infecteds at the peak of infection increases (or decreases) by a 1% increase of *x*. For instance, 〈*ϵ*_*p*_〉 = 0.27 min. means that, if we increase *p* by +1%, the peak of infection rises by 0.27%. It is important to highlight the difference between *p* and *γ* or *r*. The only free parameter of the model is *p*, while *γ* and *r* are taken from the literature. If the literature reports an inaccurate value of *γ* or *r*, this can be counterbalanced (to a certain degree) when we fit *p* to the real data. For example, if the reported value of *r* were 2.2 m instead of 2 m, we would fit the data with *p* = 3.7 × 10^−3^ min^−1^ instead of *p* = 5 × 10^−3^ min^−1^, maintaining the same accuracy of the simulation (figure 22*e*).

Finally, since the model is stochastic, we repeat the business-as-usual scenario seven times using different seeds of the random number generator. This gives slightly different results (figure 22*f* ). At each time step, we calculate the standard deviation. The maximal standard deviation is 0.94%. Therefore, at any given time, the variation due to the stochastic nature of the model is within 1%. Table 3 shows that the dependencies are mostly linear, indicating that the model is robust at least in the range of applicability of the analysis (±10%).

### (j) Putting all together 2: simulating the city of Bogotá, Colombia

**Figure 23.**
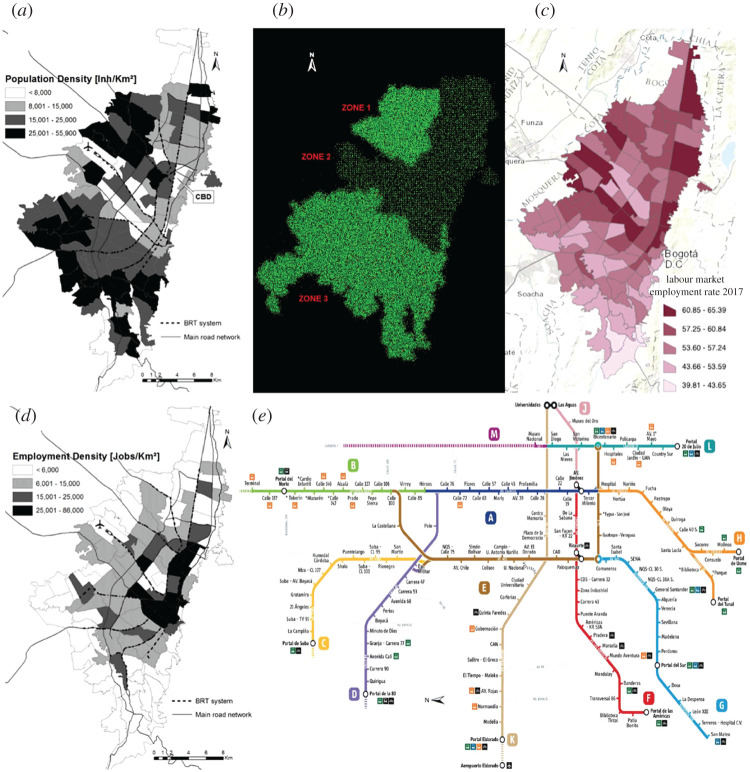
Population distribution in Bogotá [33] (*a*), density distribution in the model (*b*), occupation distribution [34] (*c*), employment distribution [33] (*d*) and Bogotá bus network (*e*). For graphical reasons, the bus network is oriented with the north pointing left.BRT, bus rapid transit; CBD, central business district. (Online version in colour.)

Bogotá has 8.3 million inhabitants. The population is distributed very unevenly (figure 23*a*). In the models, we divide the city into three zones (figure 23*b*): zone 1 with density 20 000 inhabitants per km^2^, zone 2 with density 5000 inhabitants per km^2^ and zone 3 with density 20 000 inhabitants per km^2^. This results in a virtual population of 8 292 632: 1 480 928 in zone 1; 1 115 416 in zone 2 and 5 710 748 in zone 3. The average household size is 3.9, which is handled as in §2g. The background mobility (0.7 km day^−1^) is estimated from [35]. Data on age distribution and percentage of the employed population within age groups are taken from [36,37] and managed as in §2i. Modelling transmissible diseases in Bogotá also requires the socioeconomic structure of the city to be considered. As figure 23*c* shows, the work-related activities in Bogotá are concentrated in zone 2 [33], where there are more than 785 000 enterprises registered. The majority, 96.9%, are micro-enterprises (10 employees or fewer), 2.3% are small enterprises (11–50 employees), 0.53% are medium-sized enterprises (51–200 employees) and 0.24% are big enterprises (more than 200 employees). As a first approximation, we consider all employers to be concentrated in zone 2, while zones 1 and 3 are modelled as residential districts. The occupation rate also differs in the three zones (figure 23*d*); we assume 55% occupation rate in zone 1, 60% in zone 2 and 40% in zone 3.

**Figure 24.**
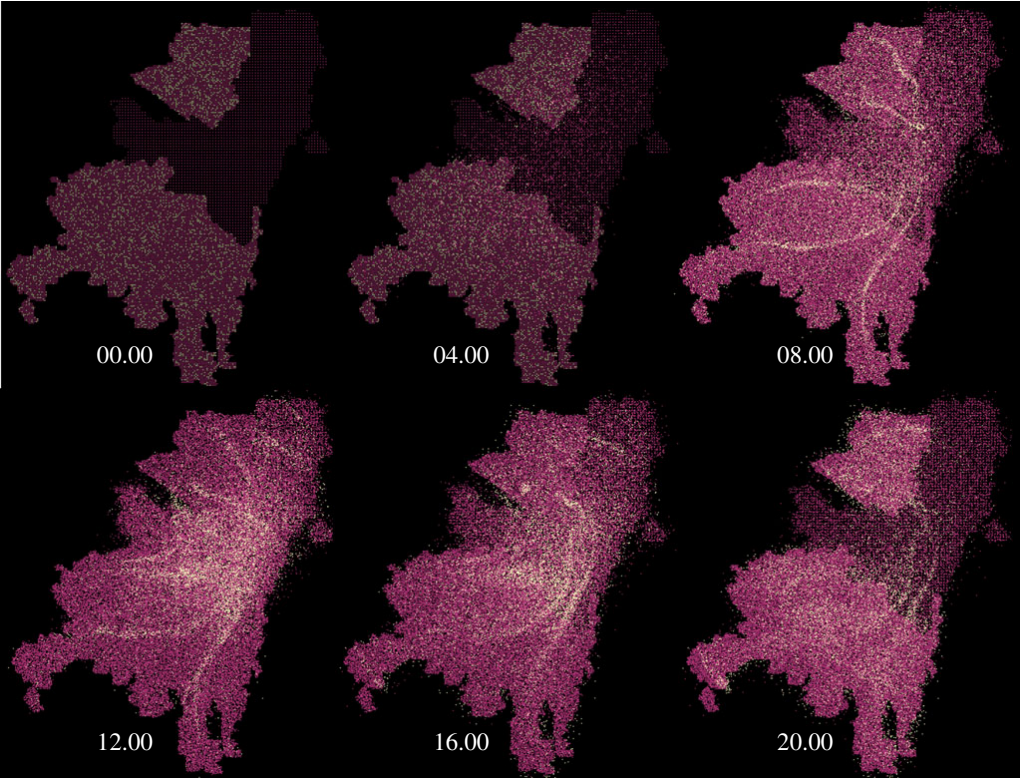
Instantaneous position of individuals taking the bus during the day (light particles) varying with time. For graphical reasons, only 1% of the population is shown. (Online version in colour.)

Because of the city’s socioeconomic structure, a large part of the population commutes every day from zones 1 and 3 to zone 2. Around 40% of commuters use the bus network (figure 23*e*), which accounts for around 2.47 million daily trips [38]. We use the technique described in §2h to model the bus network. Since we are interested in the flow from zones 1 and 3 to zone 2, we only account for routes A, B, C, D, F, G and H (figure 23*e*). The number of people using each route and the time of the day when they use the bus is taken from statistics [38]. Figure 24 shows the bus network in the model: the light dots represent the positions of individuals that use the bus at a certain point of the day.

**Figure 25.**
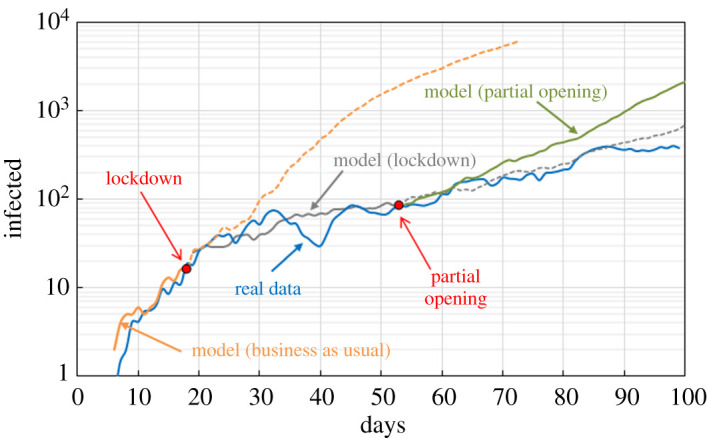
Comparison between real data (7 day moving average) and model output. (Online version in colour.)

The model is applied to the COVID-19 infection in Bogotá. Simulations are carried out with LAMMPS on Athena Midlands+ HPC. Each simulation uses 16 Xeon E5-2680v4 processors with 28 cores each and takes around 80 hours. The value *p* = 7.7 × 10^−4^ min^−1^ is derived from the pre-lockdown (‘business as usual’) real data; *r* and *γ* are considered a feature of the diseases and are the same for both Birmingham and Bogotá. Electronic supplementary material, video 5 shows the evolution of the infection in Bogotá under the ‘business as usual’ scenario. Every frame of the video represents 6 hours; for graphical reasons, only 1% of the particles is shown. Figure 25 compares the model with real data. The lockdown was implemented on 27 April 2020, the estimated number of people circulating during the lockdown was 5 × 10^5^ [39], which is consistent with the data from the Google Mobility Report [32]. According to the Mobility Report, the drop in mobility during the lockdown for retail and recreation was around 80% and around 70% for workplaces. Based on these data, we reduce the background mobility and the targeted mobility by the same amount. The bus network was closed and, therefore, not implemented in the lockdown model. On 11 May 2020, the lockdown was partially lifted and the estimated number of people circulating was 2.5 × 10^6^ [39]. The Google Mobility Report gives a reduction (with respect to the ‘business as usual’ scenario) of 59% for retail and recreation and 54% for workplaces. According to the bus operator [38], the bus service was working at 35% of its capacity. Therefore, in the ‘partial opening’ model, we reduce the background mobility by 59%, the targeted mobility by 56% and the number of people using the bus by 65%.

The model works well for the ‘business as usual’ scenario, and the lockdown, but tends to overestimate contagion when simulating reopening after the lockdown. A possible explanation is that, after lockdown is lifted, people tend to be more careful about social distancing. Interestingly, the lockdown model works well also for the reopening, but it is just a fortuitous circumstance. The percentage of infected population in Bogotá is lower than that in Birmingham. If we compare the real data before the lockdown, we can see that the number of infections is around four times higher in Birmingham. This may not be the real difference but could depend on the number of tests carried out in the two cases. More tests imply a higher detection rate, and this factor is automatically incorporated in the value of the free parameter *p* fitting the data. Therefore, different scenarios should only be compared in relative terms and with respect to the same city. Moreover, if the rate of testing in one city changes considerably, *p* should be recalculated.

**Figure 26.**
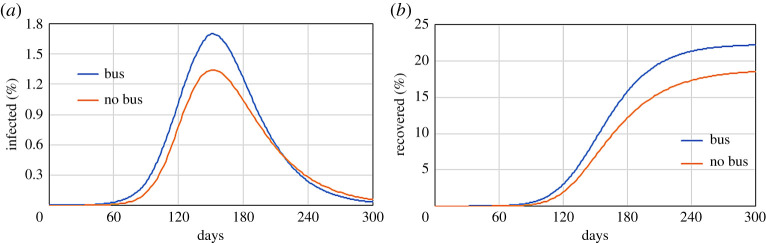
Time evolution of (*a*) infected and (*b*) recovered populations for Bogotá comparing the ‘business as usual’ and the ‘no bus’ scenarios. (Online version in colour.)

The model can assess various features of the city that influence people’s mobility. Since the public transport system is considered one of the main sources of contagion in Bogotá, we can compare the ‘business as usual’ scenario with a ‘no bus’ scenario, where commuters do not use the bus to move from home to the workplace. Since in the model 40% of commuters use the bus, in the ‘no bus’ scenario we need to set the background mobility of the individuals who normally take the bus. If we confer a high mobility to these individuals, their potential for spreading the disease becomes higher rather than lower. In this case in fact, instead of just commuting from home to work, they spend the whole day wandering around and infecting other individuals. In the simulation, we assume that this part of the population stays at home. This is somehow arbitrary, but this is a hypothetical scenario and there are no Google Mobility Report data available to accurately set this parameter in the model. Figure 26 shows that, at the peak of infection, the two scenarios differ by ~0.36%, which is around 30 000 people.

**Figure 27.**
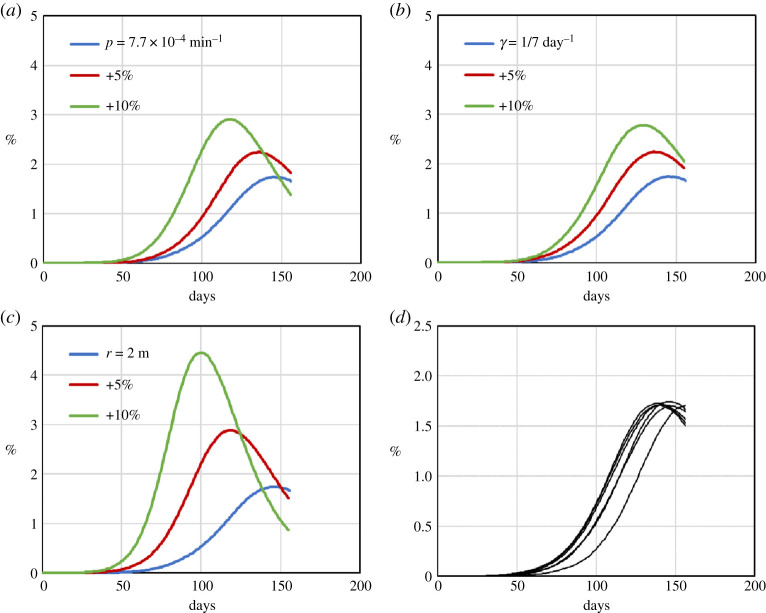
Sensitivity and statistical analysis of the Bogotá model. Effect of a +5% and +10% change in *p* (*a*), in *γ* (*b*) and in *r* (*c*). Six simulations are carried out with different seeds of the random number generator (*d*). (Online version in colour.)

As for the Birmingham model, we conclude this section with the sensitivity and statistical analysis of the model (figure 27).

Since Bogota’s population is 10 times larger, the calculations are considerably more expensive. Simulations were carried out on the ARCHER UK National Supercomputer with 1200 cores and, to save computational time, 22 hours wall time. In this way, the simulation stops around the peak of infection (156 days), which is the point required for calculating *ϵ*_*x*_ (table 4). To avoid the peak going above 156 days, instead of carrying out the simulation for ±10% increments, we looked at variations of +5% ($\epsilon_{x}^{5}$) and +10% ($\epsilon_{x}^{10}$). Finally, we repeated the ‘business as usual’ scenario six times using different seeds of the random number generator. This brings a distribution of profiles (figure 27*d*) with maximal standard deviation 0.24%.

**Table 4.** Values of $\epsilon_{x}^{5}$ and $\epsilon_{x}^{10}$ for the parameters *p*, *γ* and *r* for the Bogotá model approximated to the second decimal place.

	$\epsilon_{x}^{5}$	$\epsilon_{x}^{10}$
*ϵ*_*p*_ (min)	0.12	0.11
$\epsilon_{\gamma }$ (day)	0.10	0.10
*ϵ*_*r*_ (m^−1^)	0.23	0.27

## 3. Conclusion

This study introduces DE: a modelling framework for simulating the spreading of infectious diseases within cities or countries. We generate digital copies of Birmingham in the UK and Bogotá in Colombia, replicating their geography, infrastructure and population. The daily activities of the virtual inhabitants and the spread of the disease are simulated for several months with a time resolution of 1 minute. Simulations accurately reproduce the COVID-19 data for Birmingham and Bogotá both before and during lockdown. By simulating the mobility, interaction and potential for contagion of millions of digital individuals, our computer models are less reliant on data and have higher forecasting power than traditional epidemiological models. Except for one adjustable parameter calculable during the pandemic early stages, the model is derived from ’hard data’, i.e. a city’s topography, population statistics and Google Mobility Reports.

Despite this, we believe that DE has not achieved its full potential yet and can be improved in several directions. (i) The current model only classifies the population into susceptible, infected and recovered (SIR). More accurate classifications such as SEIRS (which accounts for ‘exposed’), SIRC (which accounts for ‘carriers’) or SIRS (which accounts for ‘reinfected’) can be introduced to improve the model. (ii) The spatial fidelity to real-world topologies can be refined to account for the real location of every household, workplace, school, bus/train station and other landmarks in the city. (iii) Several statistics and census data can be combined to derive more granular commuting patterns, which sometimes implies resolving inconsistencies in the available data [2]. (iv) Specific aspects not directly addressed at this stage (e.g. in-hospital transmission, care home transmission, etc.) can be added to the model. Finally, the numerical algorithm can be implemented on graphics processing units (GPUs) to further increase its performance. DE’s algorithm is derived from MD, where atoms are replaced by individuals. Today, GPU-accelerated MD simulations can handle billions, and even trillions, of atoms. In principle, our model could be scaled up to account for the entire human population. The proposed framework is not limited to Birmingham or Bogotá but can be adapted to any other city or region in the world. This would certainly require time and resources, but it could occur within a modular framework where researchers around the world adapt DE to other cities and regions that are gradually interconnected to cover the entire planet.

## Supplementary Material

Video 1

## Supplementary Material

Video 2

## Supplementary Material

Video 3

## Supplementary Material

Video 4

## Supplementary Material

Video 5

## Data accessibility

The code used for the simulations is freely available under the GNU General Public License v3 and can be downloaded from the University of Birmingham repository http://edata.bham.ac.uk/545/.
